# Supplementary material for: Shared and Distinct Mechanisms of UBA1 Inactivation Across Different Diseases
Source: bioRxiv. 2023 Oct 10:2023.10.10.561769. Preprint. [Version 1] doi: 10.1101/2023.10.10.561769 (PMC10592724; doi:10.1101/2023.10.10.561769)
Supplement: Supplement 2 [file NIHPP2023.10.10.561769v1-supplement-2.pdf]

bioRxiv preprint doi: https://doi.org/10.1101/2023.10.10.561769; this version posted October 10, 2023. The copyright holder for this preprint (which was not certified by peer review) is the author/funder. This article is a US Government work. It is not subject to copyright under 17 USC 105 and is also made available for use under a CC0 license.

Supplementary Figures:

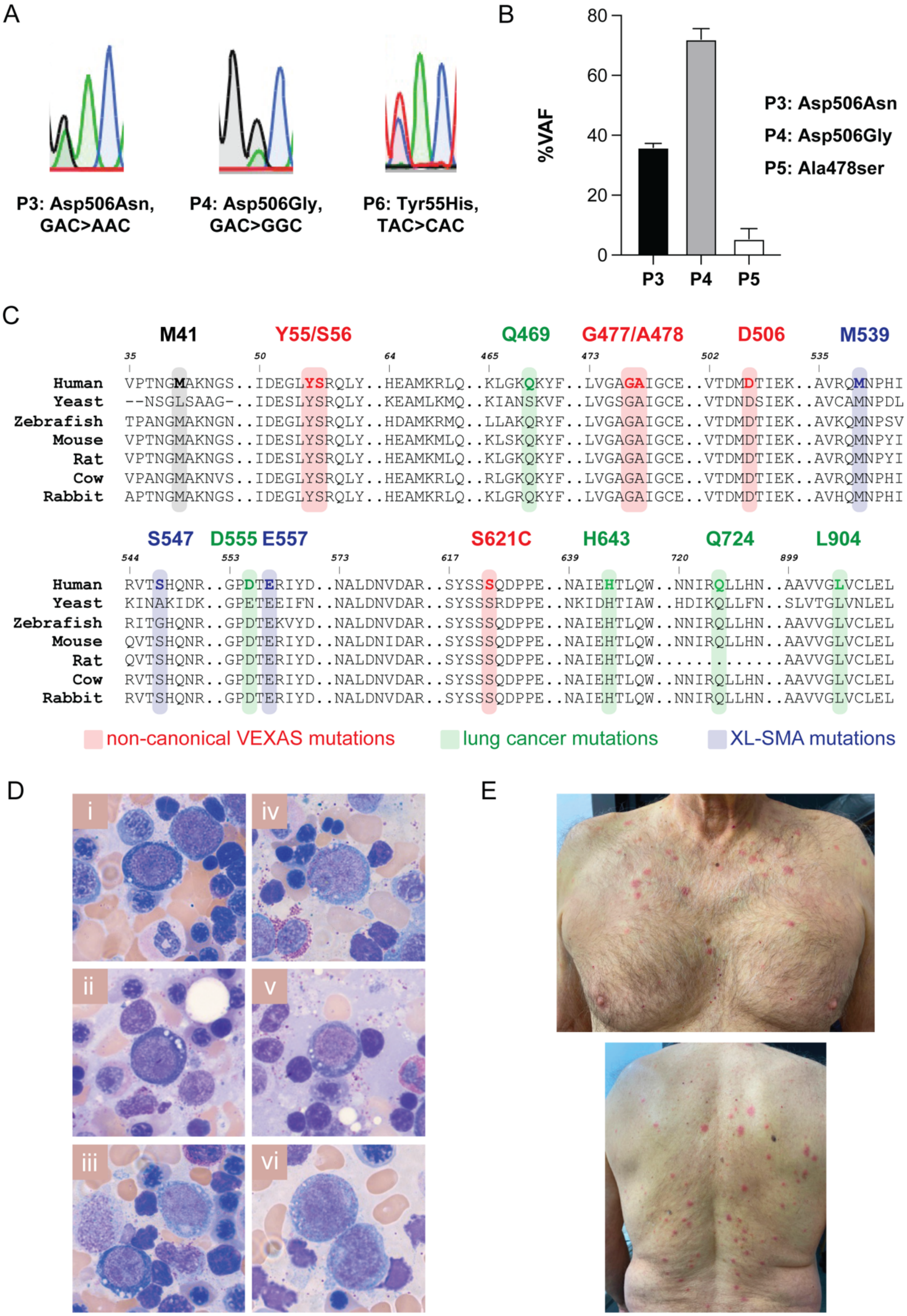

Supplementary Figure 1: Identification and clinical phenotypes of novel non-pMet41 VEXAS mutations. A) Sanger sequencing confirming novel variants. **B**) Digital

bioRxiv preprint doi: https://doi.org/10.1101/2023.10.10.561769; this version posted October 10, 2023. The copyright holder for this preprint (which was not certified by peer review) is the author/funder. This article is a US Government work. It is not subject to copyright under 17 USC 105 and is also made available for use under a CC0 license.

droplet PCR (ddPCR) confirmation for novel variants in P3, P4, P5. **C**) Conservation of protein sequence for UBA1 **D**) Cytoplasmic vacuoles were seen in a subset of the proerythroblasts (Panel i, ii, iii) and promyelocytes (Panel iii, iv, v, vi) from P6, **E**) Sweets syndrome in P6.

bioRxiv preprint doi: https://doi.org/10.1101/2023.10.10.561769; this version posted October 10, 2023. The copyright holder for this preprint (which was not certified by peer review) is the author/funder. This article is a US Government work. It is not subject to copyright under 17 USC 105 and is also made available for use under a CC0 license.

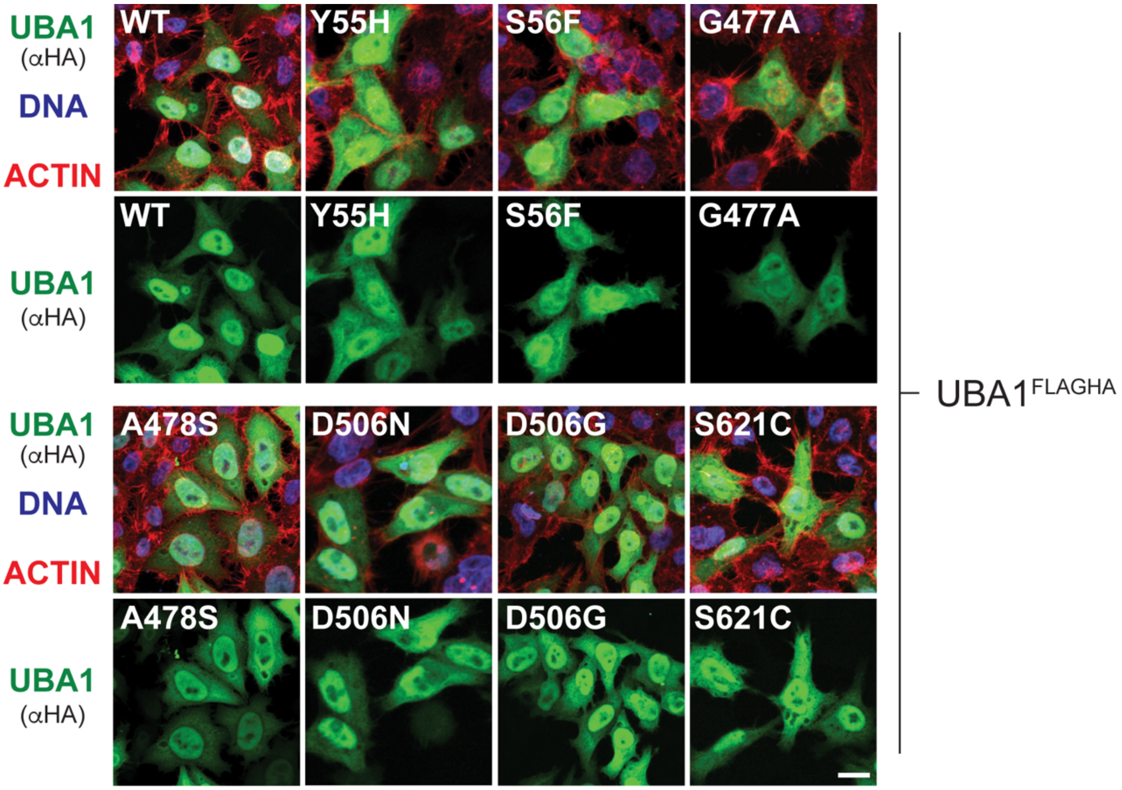

Supplementary Figure 2: Non-canonical VEXAS mutations do not cause any obvious changes in UBA1 subcellular localization. HeLa cells were transfected with indicated UBA1$^{FLAG-HA}$ variants, fixed after 72h, and subjected to immunofluorescence microscopy using antibodies against HA and phalloidin and Hoechst to visualize ACTIN and DNA, respectively. Scale Bar = 20μm.

bioRxiv preprint doi: https://doi.org/10.1101/2023.10.10.561769; this version posted October 10, 2023. The copyright holder for this preprint (which was not certified by peer review) is the author/funder. This article is a US Government work. It is not subject to copyright under 17 USC 105 and is also made available for use under a CC0 license.

A

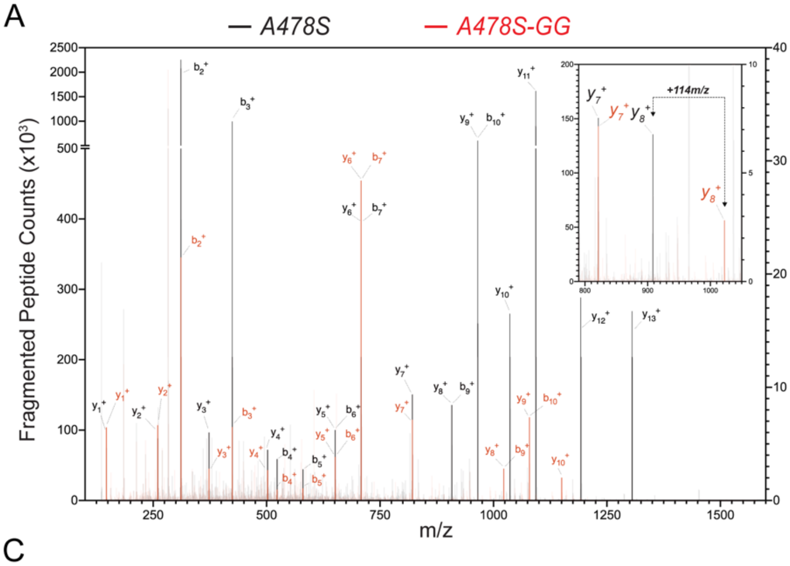

B

Peptide Fragments (1+)	A478S	A478S$_{Oxy}$	Δm/z
y_{13}	1305.69	n/a	n/a
y_{12}	1192.61	1306.65	114.04
y_{11}	1093.54	1207.58	114.04
y_{10}	1036.52	1150.56	114.04
y_9	965.48	1079.52	114.04
y_8	908.46	1022.50	114.04
y_7	821.43	821.43	0.00
y_6	708.34	708.34	0.00
y_5	651.32	651.32	0.00
y_4	502.32	502.32	0.00
y_3	373.28	373.28	0.00
y_2	260.20	260.20	0.00
y_1	147.11	147.11	0.00

C

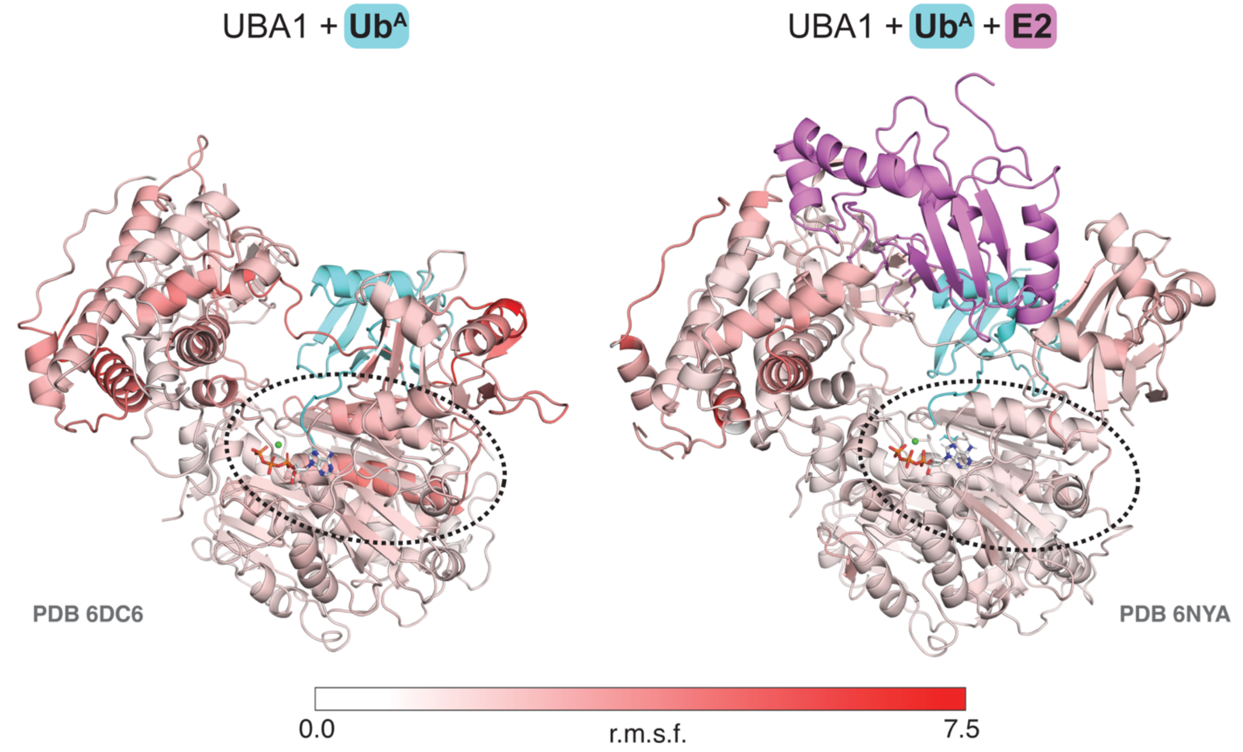

Supplementary Figure 3: UBA1 p.A478S forms an aberrant oxyester at the mutation site. A) Annotated MS/MS spectrum of A478S peptide with (red) and without (black) diGly remnant. Insert highlights the difference of 114 Da, indicative of the diGly remnant. **B**) Table summarizing the masses of fragments of the unmodified and ubiquitylated A478S peptide, pinpointing the diGly remnant (Δm/z 114.04Da) on S478. **C**) Side-by-side ribbon diagrams of UBA1 bound to ubiquitin (cyan) and ATP (indicated as a stick diagram with carbon, oxygen, nitrogen, and phosphorus in grey, red, blue, and orange) following 100 ns of molecular dynamics in the absence (left, PDB: 6DC6) or presence (right, PDB: 6NYA) of E2 enzyme (Ubc3, purple). The per residue r.m.s.f.

bioRxiv preprint doi: https://doi.org/10.1101/2023.10.10.561769; this version posted October 10, 2023. The copyright holder for this preprint (which was not certified by peer review) is the author/funder. This article is a US Government work. It is not subject to copyright under 17 USC 105 and is also made available for use under a CC0 license.

(root mean square fluctuation) value is indicated as a red gradient (scale bar). A black dashed circle highlights the region where ubiquitin, ATP, and Mg^{2+} (green sphere) bind.

bioRxiv preprint doi: https://doi.org/10.1101/2023.10.10.561769; this version posted October 10, 2023. The copyright holder for this preprint (which was not certified by peer review) is the author/funder. This article is a US Government work. It is not subject to copyright under 17 USC 105 and is also made available for use under a CC0 license.

A

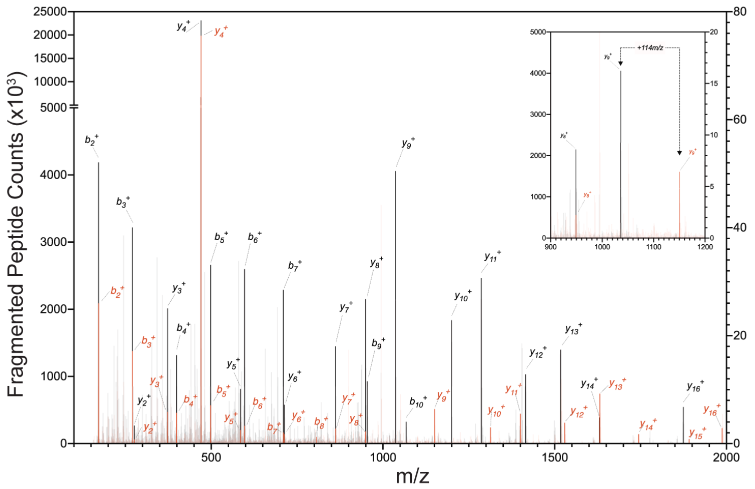

B

Peptide Fragments (1+)	S621	$S621C_{OxyS619}$	Δm/z
y_{16}	1873.80	1987.84	114.04
y_{15}	n/a	1890.79	n/a
y_{14}	1629.68	1743.72	114.04
y_{13}	1516.59	1630.64	114.04
y_{12}	1415.55	1529.59	114.04
y_{11}	1286.50	1400.55	114.04
y_{10}	1199.47	1313.51	114.04
y_{9}	1036.41	1150.45	114.04
y_{8}	949.38	949.38	0.00
y_{7}	862.34	862.34	0.00
y_{6}	713.35	713.35	0.00
y_{5}	585.29	585.29	0.00
y_{4}	470.26	470.26	0.00
y_{3}	373.21	373.21	0.00
y_{2}	276.16	276.16	0.00

C

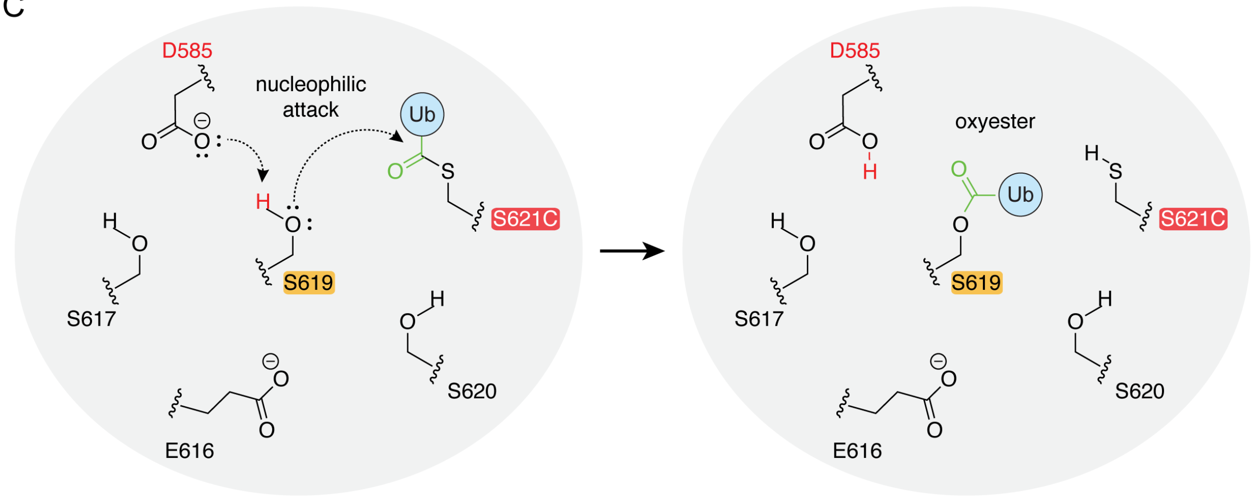

Supplementary Figure 4: UBA1 p.S621C forms an aberrant oxyester at S619 via a thioester intermediate at S621C. A) Annotated MS/MS spectrum of S621C peptide with (red) and without (black) diGly remnant. Insert highlights the difference of 114 Da, indicative of the diGly remnant. **B**) Table summarizing the masses of fragments of the unmodified and ubiquitylated S621C peptide, pinpointing the diGly remnant (Δm/z 114.04Da) on S619. **C**) Proposed catalytic mechanism for oxyester formation through deprotonation of S619 via the putative catalytic base D585 forming an oxyanion that promotes a nucleophilic attack of the thioester at S621C.

bioRxiv preprint doi: https://doi.org/10.1101/2023.10.10.561769; this version posted October 10, 2023. The copyright holder for this preprint (which was not certified by peer review) is the author/funder. This article is a US Government work. It is not subject to copyright under 17 USC 105 and is also made available for use under a CC0 license.

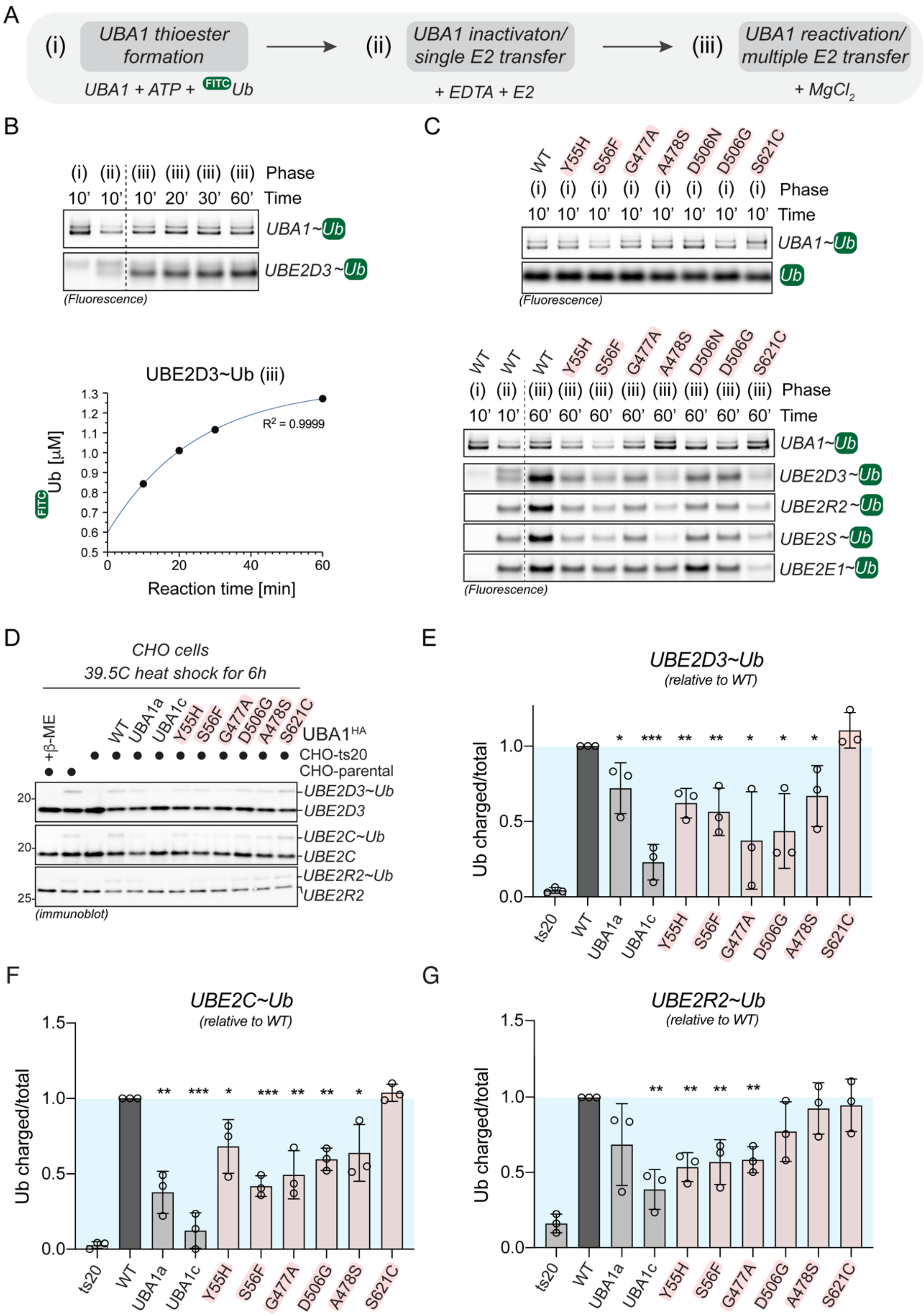

Supplementary Figure 5: Non-canonical VEXAS mutations are deficient in transferring ubiquitin to diverse E2 enzymes *in vitro* and exhibit defects in cells.
A) Schematic overview of the sequential, three-phase *in vitro* assay used to measure

bioRxiv preprint doi: https://doi.org/10.1101/2023.10.10.561769; this version posted October 10, 2023. The copyright holder for this preprint (which was not certified by peer review) is the author/funder. This article is a US Government work. It is not subject to copyright under 17 USC 105 and is also made available for use under a CC0 license.

UBA1 transthiolation. (i) Complete charging of UBA1 by incubation of 250nM UBA1b with 10μM FITC-ubiquitin and 5mM ATP (ii) Quenching of UBA1 charging and single transfer to E2 enzyme by addition of 100mM EDTA and 1μM E2 enzyme (iii) Reactivation of UBA1 charging and multi transfer to E2 enzyme by addition of 100mM $MgCl_2$ **B**) UBA1 WT was subjected to the experiment described in panel A using UBE2D3. Reactions were subjected to SDS page and analyzed by fluorescence imaging. *Upper panel*: Fluorescence scan showing UBA1 and UBE2D3 ubiquitin thioester levels after each reaction phase. *Lower panel*: UBE2D3 ubiquitin thioester levels in reaction phase (iii) were quantified and plotted against the reaction time, revealing that UBE2D3 is maximally charged after 60min. **C**) Non-canonical VEXAS mutations are deficient in E2 transthiolation *in vitro*. Indicated UBA1 proteins were subjected to the experiment described in panel A using either UBE2D3, UBE2R2, or UBE2S. Reactions were subjected to SDS page and analyzed by fluorescence imaging. *Upper panel*: Fluorescence scan showing UBA1 charging after reaction phase (i) as control. *Lower panel*: Fluorescence scan showing UBA1 re-charging and E2 transfer after reaction phase (iii). Quantifications of 3 biological replicates are shown in Figure 5F. **D**) Non-canonical VEXAS mutations are impaired in supporting E2 ubiquitin thioester levels in cells. CHO ts20 cells were reconstituted with indicated UBA1 variants and incubated at the permissive temperature for 6h, followed by immunoblotting using antibodies against indicated E2 enzymes. **E-G**) Quantification of E2 charging levels (charged/total) shown in panel D. E) UBE2D3, F) UBE2C, G) UBE2R2. n=3 biological replicates, error bars = s.d., * = $p < 0.05$, ** = $p < 0.01$, *** = $p < 0.001$, one-way ANOVA.

bioRxiv preprint doi: https://doi.org/10.1101/2023.10.10.561769; this version posted October 10, 2023. The copyright holder for this preprint (which was not certified by peer review) is the author/funder. This article is a US Government work. It is not subject to copyright under 17 USC 105 and is also made available for use under a CC0 license.

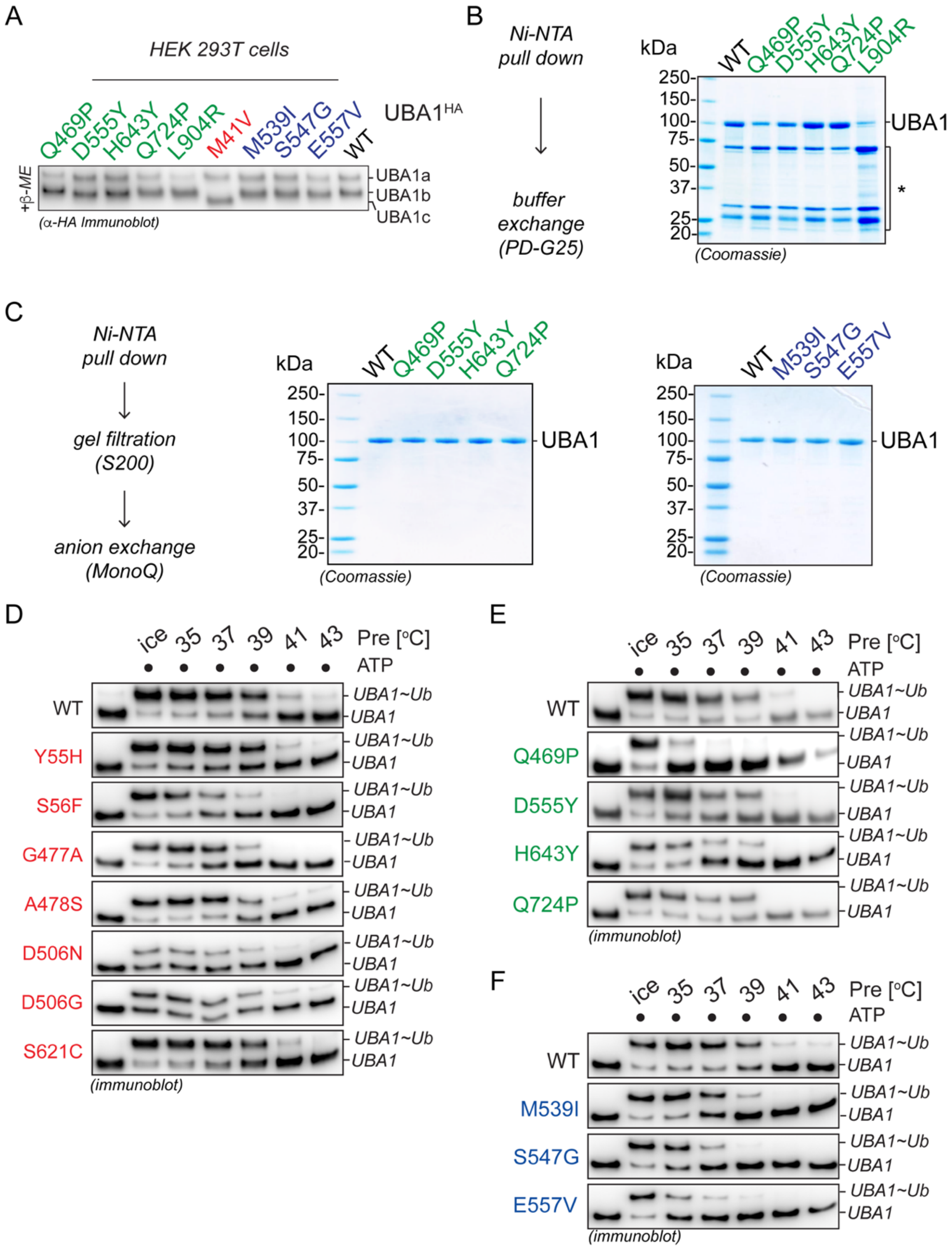

Supplementary Figure 6: SMA-causing and a subset of LCINS-associated mutations render UBA1 activity thermolabile. A) SMA-causing mutations and

bioRxiv preprint doi: https://doi.org/10.1101/2023.10.10.561769; this version posted October 10, 2023. The copyright holder for this preprint (which was not certified by peer review) is the author/funder. This article is a US Government work. It is not subject to copyright under 17 USC 105 and is also made available for use under a CC0 license.

LCINS-associated UBA1 mutations do not lead to the cytoplasmic isoform swap from UBA1b to UBA1c, as evidenced by immunoblotting of HEK 293T cell lysates transfected with the indicated UBA1 variants. **B**) Coomassie-stained SDS page gel of indicated LCINS-associated UBA1 variants expressed and purified from *E.coli* via Ni-NTA pull down followed by buffer exchange via PD-G25 columns. This protocol only yielded ~30% pure UBA1 proteins but did allow for production of small amounts of the folding-defective UBA1 p.L904R variant for ubiquitin thioester formation and transthiolation studies. **C**) Coomassie-stained SDS page gel of indicated LCINS-associated UBA1 mutants (left) or SMA-causing UBA1 mutants (right) expressed and purified from *E.coli* via Ni-NTA pull down, S200 gel filtration, and anion exchange chromatography, revealing highly protein purity. **D**) UBA1 p.S56F is the only non-canonical VEXAS mutation that renders UBA1 thiolation thermolabile. Indicated UBA1 proteins were pre-incubated at varying denoted temperatures in the absence of substrate, subjected to ubiquitin thioester formation assays on ice using 500nM UBA1, 10μM ubiquitin, and 5mM ATP, and analyzed by anti-UBA1 immunoblotting. Quantifications are depicted in Figure 6D. **E**) LCINS mutations p.H643Y and p.Q724P render UBA1 thiolation thermolabile. Indicated UBA1 proteins were treated as described in panel D. Quantifications are depicted in Figure 6E. **F**) All SMA-causing mutations render UBA1 thiolation thermolabile. Indicated UBA1 proteins were treated as described in panel D. Quantifications are depicted in Figure 6F.

bioRxiv preprint doi: https://doi.org/10.1101/2023.10.10.561769; this version posted October 10, 2023. The copyright holder for this preprint (which was not certified by peer review) is the author/funder. This article is a US Government work. It is not subject to copyright under 17 USC 105 and is also made available for use under a CC0 license.

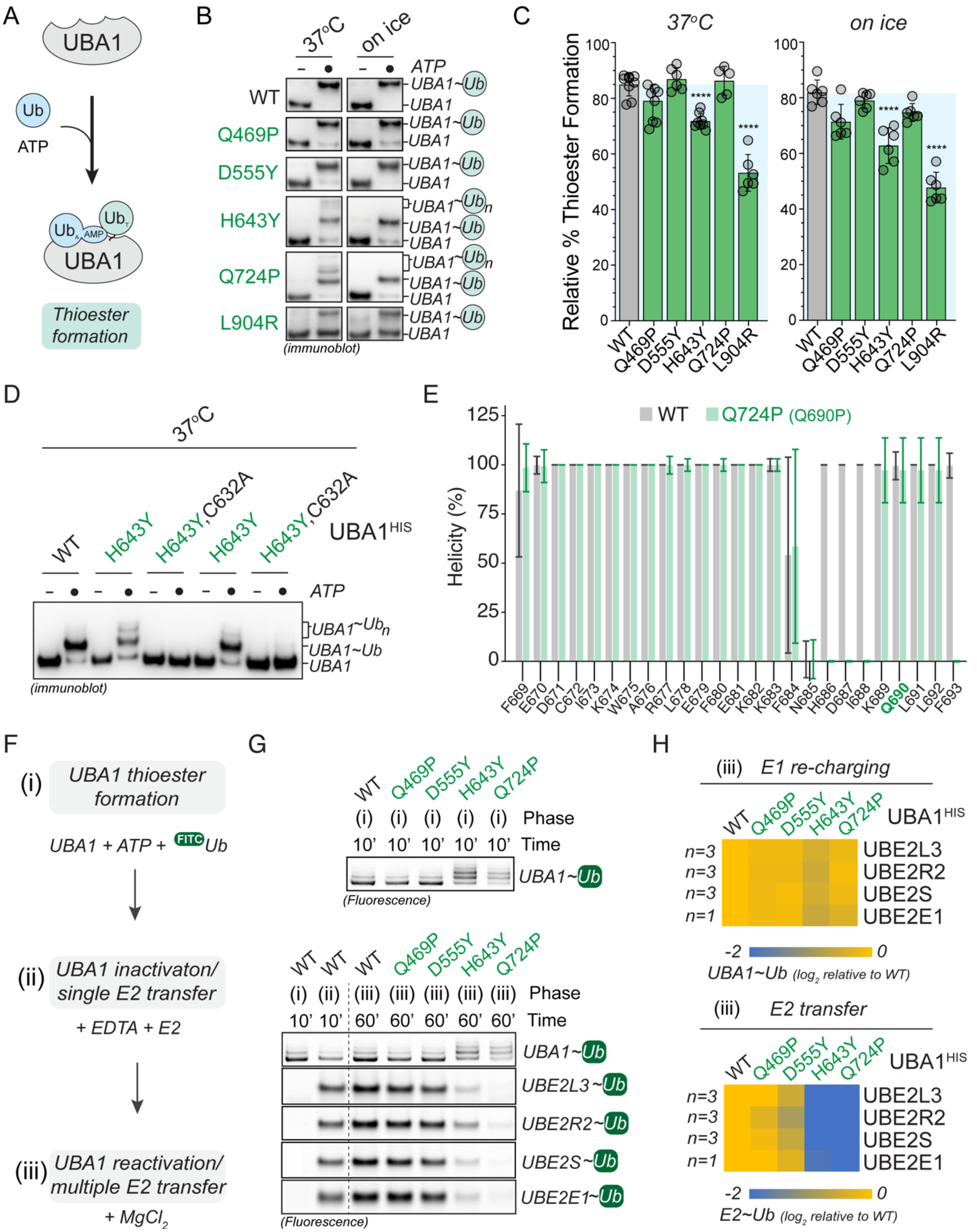

Supplementary Figure 7: LCINS-associated UBA1 p.H643Y and p.Q724P form aberrant thioesters and exhibit a bottleneck in ubiquitin transfer to E2 enzymes.
A) Schematic overview of the assay used to determine ubiquitin thioester formation

bioRxiv preprint doi: https://doi.org/10.1101/2023.10.10.561769; this version posted October 10, 2023. The copyright holder for this preprint (which was not certified by peer review) is the author/funder. This article is a US Government work. It is not subject to copyright under 17 USC 105 and is also made available for use under a CC0 license.

activity of UBA1. **B**) Non-reducing immunoblot analysis of *in vitro* ubiquitin thioester formation reactions carried out at 37°C (left panel) or on ice (right panel), revealing that UBA1 p.L904R is deficient in thiolation and that UBA1 p.H643Y and p.Q724P form aberrant thioesters at 37°C. **C**) Quantification of thioester formation (UBA1~Ub/total UBA1 signal) of the experiments shown in panel B. n = 5 – 8 biological replicates as indicated, error bars = s.d., **** = $p < 0.0001$, one-way ANOVA. **D**) Aberrant thioester formation of UBA1 p.H643Y and p.Q724P depends on the catalytic cysteine residue C632. Denoted recombinant UBA1b proteins (500 nM) were incubated with 10 μM ubiquitin and 5 mM ATP for 10 or 60 min at 37°C followed by anti-UBA1 immunoblot analysis. **E**) The average alpha helicity of residues in the helix containing Q724 (Q690) calculated over a 100 ns molecular dynamics trajectory for *S. cerevisiae* UBA1 (PDB: 4NNJ). Residues N-terminal to Q724 (Q690) in the wild-type protein (grey) maintain an average alpha helical content of 50% or greater. When the Q690P mutation (green) is modeled into the structure, the proline breaks the helix and residues (H686-I688) lose their alpha helicity. **F**) Schematic overview of the sequential, three-phase *in vitro* assay used to measure UBA1 transthiolation. (i) Complete charging of UBA1 by incubation of 250nM UBA1b with 10μM FITC-ubiquitin and 5mM ATP (ii) Quenching of UBA1 charging and single transfer to E2 enzyme by addition of 100mM EDTA and 1μM E2 enzyme (iii) Reactivation of UBA1 charging and multi transfer to E2 enzyme by addition of 100mM $MgCl_2$ **G**) LCINS mutations p.H643Y and p.Q724P but not p.Q469P or P.D555Y are deficient in E2 transthiolation *in vitro*. Indicated UBA1 proteins were subjected to the experiment described in panel F using either UBE2D3, UBE2R2, or UBE2S. Reactions were subjected to SDS page and analyzed by fluorescence imaging. *Upper panel*: Fluorescence scan showing UBA1 charging after reaction phase (i) as control. *Lower panel*: Fluorescence scan showing UBA1 re-charging and E2 transfer after reaction phase (iii). **H**) Quantifications of UBA1 ubiquitin thioester levels (*upper panel*) and E2 ubiquitin thioester levels (*lower panel*) after phase (iii) shown in panel G. E2 ubiquitin thioester levels in phase (iii) were first normalized to the total FITC signal in phase (i) and then normalized to WT. n = 1-3 biological replicates per condition as indicated.

bioRxiv preprint doi: https://doi.org/10.1101/2023.10.10.561769; this version posted October 10, 2023. The copyright holder for this preprint (which was not certified by peer review) is the author/funder. This article is a US Government work. It is not subject to copyright under 17 USC 105 and is also made available for use under a CC0 license.

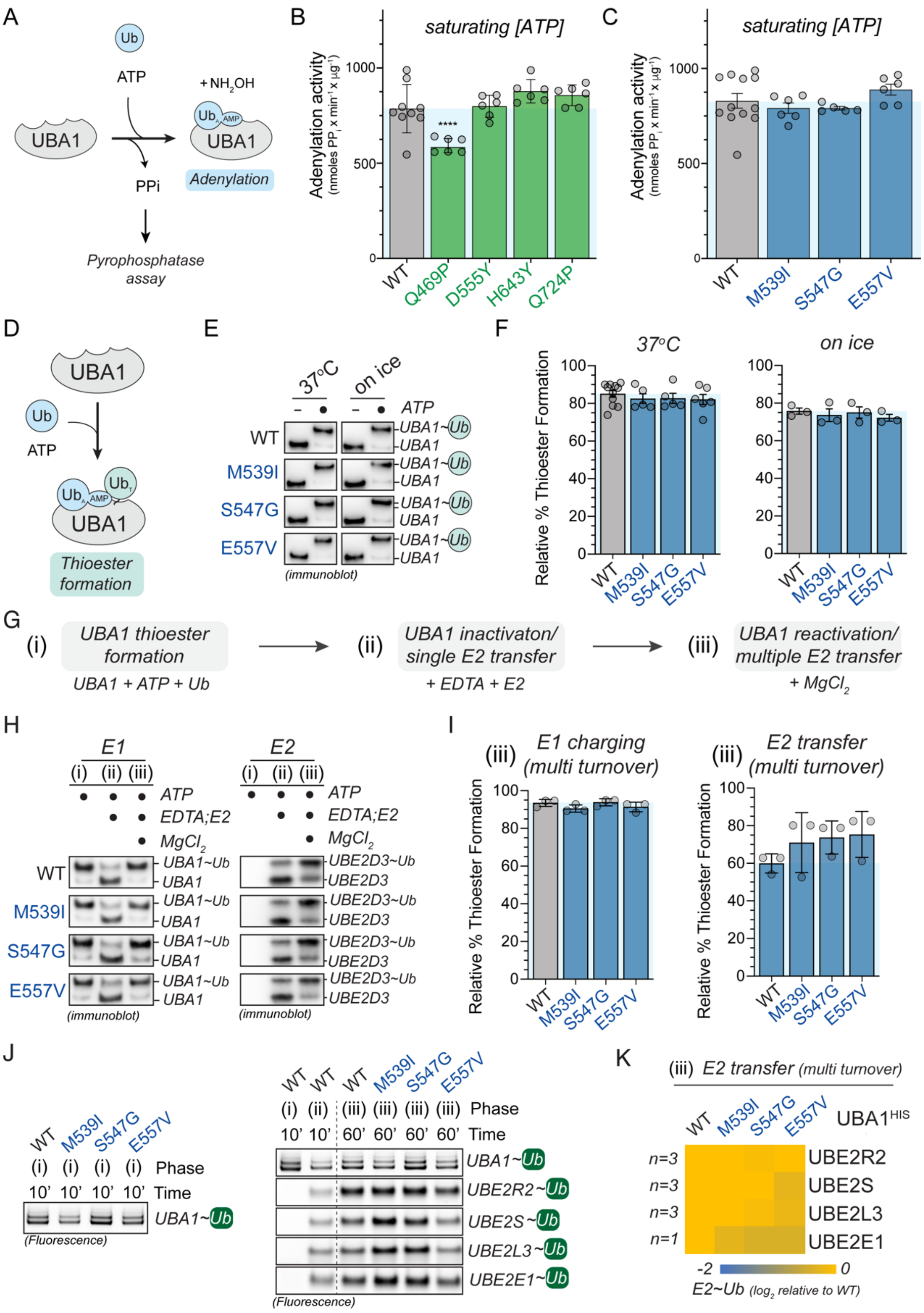

Supplementary Figure 8: Mechanistic profiling of SMA and LCINS mutations. A) Schematic overview of the *in vitro* assay used to determine the efficiency of the first

bioRxiv preprint doi: https://doi.org/10.1101/2023.10.10.561769; this version posted October 10, 2023. The copyright holder for this preprint (which was not certified by peer review) is the author/funder. This article is a US Government work. It is not subject to copyright under 17 USC 105 and is also made available for use under a CC0 license.

adenylation reaction of UBA1. The assay measures the production of pyrophosphate (PP_i) and the reaction is carried out in presence of NH_2OH to reduce the ubiquitin thioester. **B**) Amongst LCINS mutations only UBA1 p.Q469P confers a significant but small impairment of adenylation activity *in vitro* at saturating ATP and ubiquitin conditions. n = 2-3 biological replicates with 3 technical replicates each, error bars = s.d., **** = $p < 0.0001$, one-way ANOVA. **C**) SMA-causing UBA1 mutations have no obvious effect on adenylation activity *in vitro* at saturating ATP and ubiquitin conditions. n = 2-4 biological replicates with 3 technical replicates each, error bars = s.d., no significant changes observed, one-way ANOVA. **D**) Schematic overview of the assay used to determine ubiquitin thioester formation activity of UBA1. **E**) Non-reducing immunoblot analysis of *in vitro* ubiquitin thioester formation reactions carried out at 37°C (left panel) or on ice (right panel) and containing 500nM indicated UBA1b proteins, 10μM ubiquitin, and 5mM ATP, revealing that SMA-causing mutations have no obvious impact on UBA1 thiolation *in vitro*. **F**) Quantification of thioester formation (UBA1~Ub/total UBA1 signal) of the experiments shown in panel E. n = 3 – 10 biological replicates as indicated, error bars = s.d., **** = $p < 0.0001$, one-way ANOVA. **G**) Schematic overview of the sequential, three-phase *in vitro* assay used to measure UBA1 transthiolation. (i) Complete charging of UBA1 by incubation of 250nM UBA1b with 10μM ubiquitin and 5mM ATP (ii) Quenching of UBA1 charging and single transfer to E2 enzyme by addition of 100mM EDTA and 1 μM UBE2D3 (iii) Reactivation of UBA1 charging and multi transfer to E2 enzyme by addition of 100mM $MgCl_2$. **H**) SMA-causing mutations have no obvious impact on E2 transthiolation *in vitro*. Immunoblot analysis of the sequential, three-phase *in vitro* assay described in panel G using antibodies against UBA1 (left) or UBE2D3 (right). **I**) Quantifications of relative UBA1 re-charging (UBA1~Ub/total signal, left graph) and E2 thioester levels (UBE2D3~Ub/total signal, right graph) of multi turnover reactions (phase iii) depicted in panel H. n = 3 biological replicates as indicated, error bars = s.d., no significant changes detected, one-way ANOVA. **J**) SMA-causing UBA1 mutations are not defective in E2 transthiolation *in vitro*. Indicated UBA1 proteins were subjected to the experiment described in panel G using either UBE2D3, UBE2R2, or UBE2S. Reactions were run on SDS page and analyzed by fluorescence imaging. *Left panel*: Fluorescence scan showing UBA1 charging after reaction phase (i) as control. *Right panel*: Fluorescence scan showing UBA1 re-charging and E2 transfer after reaction phase (iii). **K**) Quantifications of E2 ubiquitin thioester levels after phase (iii) shown in panel G. E2 ubiquitin thioester levels in phase (iii) were first normalized to the total FITC signal in phase (i) and then normalized to WT. n = 1-3 biological replicates per condition as indicated.

bioRxiv preprint doi: https://doi.org/10.1101/2023.10.10.561769; this version posted October 10, 2023. The copyright holder for this preprint (which was not certified by peer review) is the author/funder. This article is a US Government work. It is not subject to copyright under 17 USC 105 and is also made available for use under a CC0 license.

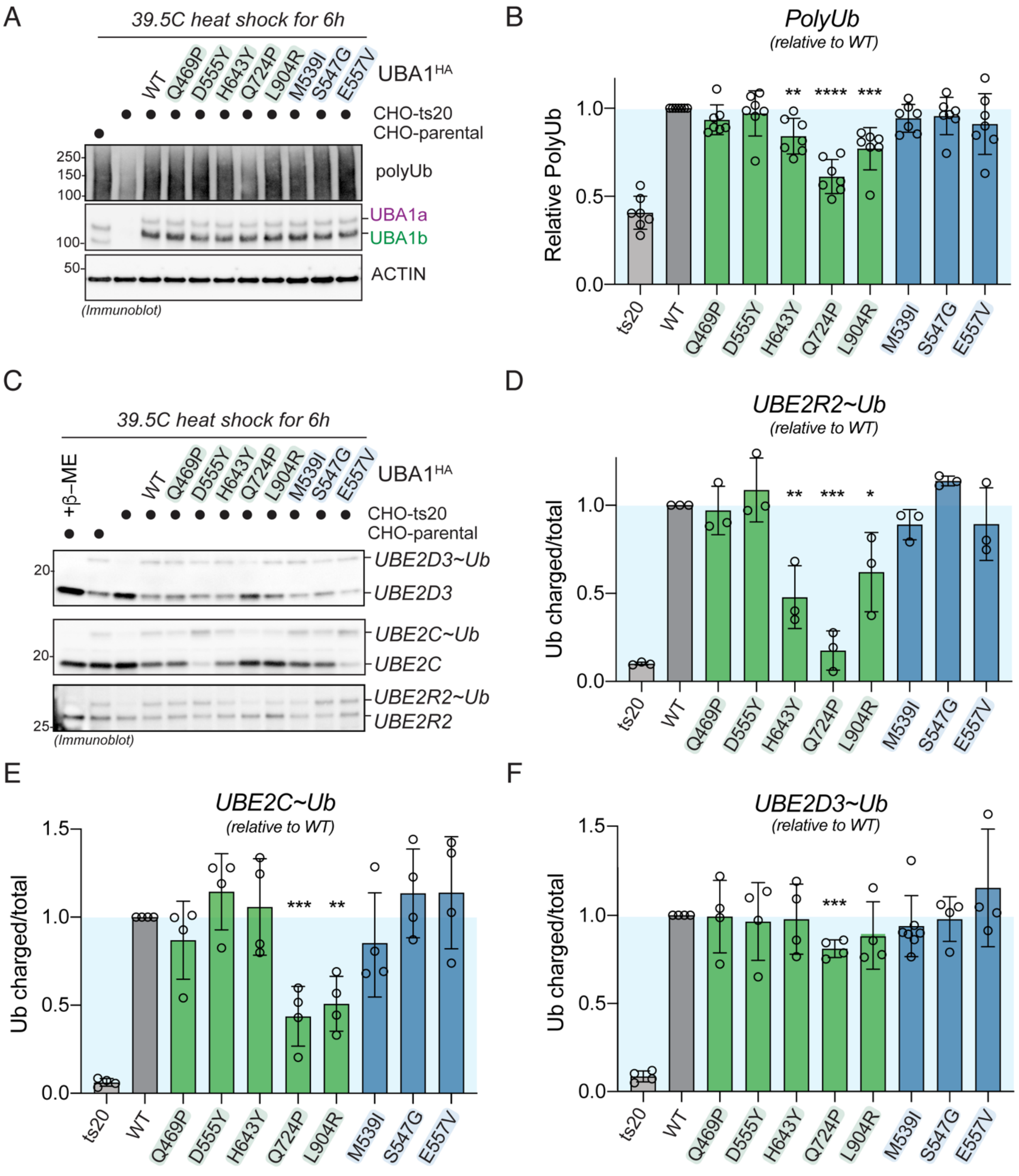

Supplementary Figure 9: Profiling of SMA and LCINS mutations in CHO cells. A) Immunoblots of CHO ts20 cells reconstituted with indicated UBA1 variants and incubated at the permissive temperature for 6h, revealing that a subset of LCINS mutations are impaired in their ability to support ubiquitylation in cells. **B**) Quantification of global polyubiquitylation levels shown in panel A. n=7 biological replicates, error bars = s.d., ** = p < 0.01, *** = p < 0.001, **** = p < 0.0001, one-way ANOVA. **C)**

bioRxiv preprint doi: https://doi.org/10.1101/2023.10.10.561769; this version posted October 10, 2023. The copyright holder for this preprint (which was not certified by peer review) is the author/funder. This article is a US Government work. It is not subject to copyright under 17 USC 105 and is also made available for use under a CC0 license.

Immunoblots of CHO ts20 cells reconstituted with indicated UBA1 variants and incubated at the permissive temperature for 6h, revealing that a subset of LCINS mutations are impaired in their ability to support E2 ubiquitin thioester levels in cells. **D-F**) Quantification of E2 charging levels (charged/total) shown in panel C. D) UBE2R2, E) UBE2C, F) UBE2D3. n=3 biological replicates, error bars = s.d., * = $p < 0.05$, ** = $p < 0.01$, *** = $p < 0.001$, **** = $p < 0.0001$, one-way ANOVA.
